# Late Relapse and Follow-up Protocols in Testicular Germ Cell Tumours: The Edinburgh Cancer Centre Experience and Review of the Literature

**DOI:** 10.4137/cmo.s321

**Published:** 2008-01-21

**Authors:** Beatrice Detti, Paul A. Elliott, Duncan B. McLaren, Grahame C.W. Howard

**Affiliations:** 1 Department of Radiotherapy, University of Florence, Florence, Italy; 2 Edinburgh Cancer Centre, Western General Hospital, Crewe Road South, Edinburgh, Midlothian, Scotland, EH4 2XU

**Keywords:** follow-up, germ cell tumors, late relapse

## Abstract

**Aims:**

To identify clinicopathological features and outcomes in patients with late relapse (LR) of testicular germ cell tumours (GCTs) in order to guide follow-up policy.

**Materials and Methods:**

The Edinburgh Cancer Centre (ECC) database identified all patients diagnosed with testicular GCT between 1988 and 2002. Of 703 patients, six relapsed more than 24 months after their initial treatment. A retrospective casenote review was performed to extract clinical, pathological, treatment and outcome data.

**Results:**

Six patients (0.85%) underwent late relapse. All patients presented initially with stage I disease and five were classified as good risk (International Germ Cell Consensus Classification, IGCCC). Median time to LR was 31 months. Two patients had previously relapsed less than 24 months from initial diagnosis. Markers at the time of relapse were normal in all patients. In all cases of late relapse disease was confined to axial lymphadenopathy. Three patients were treated with chemotherapy alone, two patients underwent surgical resection and one patient received combined treatment. All patients obtained a complete response and all remain disease free with a median follow-up of 52 months.

**Conclusions:**

The incidence of late relapse in this series is low. Chemo-naive patients with LR were successfully salvaged with chemotherapy alone and patients previously exposed to cisplatin-based chemotherapy were salvaged with complete surgical excision. The optimal length of follow-up in patients with testicular germ cell tumours is not known and practice varies widely. In this cohort of 703 patients, only one patient who relapsed was picked up by additional clinic follow-up between 5 and 10 years. Thus, on the basis of this small series, the authors suggest that follow-up after five years may not be justified.

## Introduction

Testicular germ cell tumours (GCTs) are rare, accounting for only 1% of all male cancers. However, they are the most common solid tumours in young men with a peak incidence at 25–35 years [Dearnely, 2001]. The highest incidence is in northern European males [Devesa, 1995] and approximately 1400 new cases are diagnosed annually in the United Kingdom. The worldwide incidence has more than doubled over the past 40 years for reasons that remain unclear. Testicular maldescent is a recognized aetiological factor with a relative risk (RR) of 4.8, familial testicular cancer constitutes a RR of 3–10 and ipsilateral testicular germ cell cancer results in a 25-fold increased risk of developing a second GCT in the contralateral testis [Dieckmann, 2004].

Cure rates for this disease are high although decrease with increasing stage and worsening prognostic group at presentation. [Bhatia, 2000; Miller, 1997; IGCCC 1997]. Relapse rates vary between 10% and 30%, depending on the stage of disease with most relapses occurring within 2 years, the majority of patients being salvaged with further treatment [Howard, 2005].

A minority of patients (2%–3%) will undergo a “Late Relapse” (LR), defined as metastatic disease arising two or more years after completion of primary therapy in the absence of a metachronous GCT or second non-testicular primary cancer. The time to late relapse has been reported to range from 2 to 32 years with a median of approximately 6 years [IGCCC 1997]. Although late relapses of GCT have been reported in the chest, neck, pelvis, brain, and liver, the most common site is the retroperitoneum, regardless of initial stage, clinical presentation, or prior treatment [Baniel, 1995]. Alpha-fetoprotein (αFP) is the most frequently raised tumour marker, though frequently αFP, beta human chorionic gonadotrophin (β-hCG) and lactate dehydrogenase (LDH) are all normal.

We have previously published the management and outcomes of a cohort of 703 patients diagnosed at the ECC between 1988 and 2002 with Germ Cell Tumours of the testis [Howard, 2005]. In order to develop guidance on longterm follow-up scheduling we studied the further management and outcomes of a subgroup of these patients who, after achieving a complete response, subsequently relapsed after a gap of 24 months or more.

## Patients and Methods

The ECC database identified all patients diagnosed with testicular GCT between 1988 and 2002 who relapsed at or more than 24 months after completion of primary therapy (orchidectomy with or without chemotherapy or radiotherapy). Casenotes were examined to extract clinical, pathological, mode of treatment and outcome data.

## Results

From 1988 to 2002, 703 patients presented to the ECC with testicular GCT. Six patients (0.85%) relapsed at or more than 24 months after their initial treatment.

### Features at initial presentation of primary testicular GCT (Table 1)

The median age of the six patients at the time of their initial diagnosis was 39 years (range; 24 to 51). All patients presented initially with stage I disease (Royal Marsden Hospital staging classification [Peckham, 1971]) and five were in the IGCCC good prognostic group [IGCCC 1997]. One patient was in the intermediate prognostic group. All patients underwent initial trans-inguinal orchidectomy. Histopathological examination of the primary tumour revealed malignant teratoma intermediate (MTI) in three patients, mixed seminoma/non-seminomatous germ cell tumour (GCT) in one patient and seminoma in two patients.

At initial diagnosis serum markers were normal in three cases. The β-hCG was elevated in one patient (11150 U/L), and the αFP elevated in two patients (115 and 387 kU/L).

Of the two patients with seminoma, one was treated with para-aortic strip radiotherapy (30 Gy in 15 fractions over three weeks) as per the Edinburgh Cancer centre protocol at that time. The second patient could not be treated with radiotherapy because of severe thoracolumbar kyphosis and thus went into a surveillance protocol. Four patients with nonseminomatous germ cell tumours (NSGCT) were followed up on our surveillance protocol.

### Patterns of relapse

Of the whole cohort of 703 patients diagnosed with primary testicular GCT, 446 (63%) had seminoma and 257 (37%) non-seminoma. 26% initially presented with metastatic disease. Overall, 42 patients relapsed within 2 years of completion of initial treatment. The management and outcomes of these patients have been described previously [Howard, 2005]. Two of these 42 patients (4.7%) subsequently relapsed a second time and are described here. (Table 1, patients 5 and 6).

In both cases the initial presenting pathology was MTI and the site of early relapse was the retroperitoneum. Both patients were asymptomatic at the time of initial relapse and the diagnosis was made by routine surveillance CT scanning.

Treatment of these two patients at first relapse involved 4 cycles of chemotherapy with bleomycin, etoposide and cisplatin (BEP), followed by surgical excision of residual masses with retroperitoneal lymph node dissection (RPLND). Histopathology revealed teratoma differentiated (TD) in both cases. Complete surgical excision was identified pathologically, and biochemical and radiological complete response (CR) was confirmed.

For the six patients patients who relapsed more than 24 months after initial diagnosis the median time to relapse was 31 months (range; 24 to 135); in two patients relapse occurred more than ten years after primary treatment. (Table 1).

Three late relapses were identified during scheduled follow up, in two patients retroperitoneal disease was identified on CT scanning, and in one a chest X ray detected a mediastinal mass. Three patients required an unscheduled clinic attendance for symptomatic disease whilst still on routine follow up. All three had cervical lymphadenopathy and a staging CT scan confirmed additional retroperitoneal disease in one patient. Serum tumour markers were normal in all patients at the time of LR including three patients who were marker positive at primary diagnosis.

### Management of late relapse (Table 2)

Three patients who were chemotherapy naïve were treated with chemotherapy alone, (4 cycles of BEP in one patient and 4 cycles of EP in two patients). All achieved a radiological complete response and have not required further treatment. Two patients underwent a surgical resection which revealed mature teratoma undifferentiated, one containing a small focus of adenocarcinoma. In both cases resection was considered to be complete and neither patient received post surgical chemotherapy. In the third patient a resected neck node confirmed metastatic seminoma and he subsequently received four cycles of EP chemotherapy.

After treatment all six patients obtained a complete pathological, biochemical and radiological response. All patients remain alive and disease free with a median follow up of 52 months (range 20–136 months).

## Discussion

Orchidectomy is curative in many early stage testicular cancers. The introduction of platinum-based chemotherapy in the late 1970s dramatically improved the outcome for patients with metastatic disease. 10%–30% of patients relapse within the first two years following primary management and many are salvaged with surgery with or without chemotherapy. Historically a complete remission, lasting more than two years, was felt to represent a cure. However, more recently it has become apparent that in a small number of patients (2%–3%) late relapse may occur up to thirty years after initial treatment [IGCCC 1997]. Late relapses may represent a distinct clinical subgroup of testicular GCTs. Primary testicular GCTs are usually rapidly progressive and respond well to chemotherapy; however, late relapses frequently respond less well to platinum-based chemotherapy, particularly if initial treatment included systemic therapy [George, 2003].

### Review of literature

A Pubmed search of the current literature on late relapse identified nearly 400 cases reported in six retrospective studies [Baniel, 1995; Gerl, 1997; Shahidi, 2002; Dieckmann, 2002; George, 2003; Ronnen, 2005] (Table 3) and several smaller series [Terebelo, 1983; Ravi, 1997; Carver, 2005; Geldart, 2006].

Dieckmann et al. [Dieckmann, 2005] retrospectively analyzed 122 patients with late relapse, from 24 institutions in Germany. The median time to late relapse was 49.5 months. 61% of LRs were detected incidentally during routine follow up and 12% of all patients with late relapse in this series had previously presented with an earlier relapse. 60% of patients received surgery alone or as a component of their treatment of LR and 63% remained free from subsequent recurrence with an unreported length of follow-up.

Indiana University Medical Centre [Baniel, 1995] reported 81 late relapses from 1979 to 1992, of which 35 occurred in patients who originally presented with stage I disease. They showed a disease-free survival rate of 25.9% after a median follow up of 4.8 years. In this report only 2 patients treated with chemotherapy alone remained disease free at 12 and 102 months of follow up, compared with 68.7% of patients treated with surgical resection, at a median follow up of 14 months. George et al. [George, 2003] reviewed 83 further cases of LR, treated at the same centre from 1993 to 2000. 41% of patients treated with surgery alone remained free from subsequent relapse, with a median follow up of 37 months, whilst only 15.6% of men who received chemotherapy alone remained continuously disease-free at a median follow up of 44 months.

Shahidi et al. [Shahidi, 2002] identified 53 patients with LR. In a multivariate analysis, positive tumour markers at initial presentation and the presence of TD in post-chemotherapy surgical specimens were predictive of late recurrence. They suggested that follow-up to detect recurrence may not be required after 5 years, except in those presenting with metastatic non-seminomatous germ cell tumours of the testis where lifelong follow-up was recommended.

At MSKCC [Carver, 2005], with a median follow-up of 42 months, 21 of 50 patients (42%) remained disease free following surgical resection (with or without prior chemotherapy) for late relapse. Predictors of survival after therapy for late relapse of GCT included complete surgical resection, single site of disease and histological finding of TD in the resection specimen.

Ronnen et al. [Ronnen, 2005], recently presented 29 patients with non-seminomatous GCTs treated with chemotherapy at LR. In this series the median time to late relapse was 10.5 years and the median survival was 23.9 months. In 72% of cases LR was detected by symptomatic presentation. With a median follow-up of 50.6 months, 7 patients achieved a sustained complete response, six of them had received surgical resection of residual masses.

Gerl et al. [Gerl, 1997] reviewed 25 patients with LR and reported a disease-free survival rate of 36% at a median follow up of 38 months. At diagnosis of LR serum αFP was increased in 67% of patients. In this series patients previously presenting with an early relapse had a cumulative risk of late relapse of 9.4% at 5 years and 29% at 10 years.

Patients who relapse late, either on surveillance or after initial treatment appear to represent a subgroup with risk factors being a previous relapse and TD in post-treatment resected masses. The median time to LR is approximately 6 years. Most series demonstrate that surgical resection is associated with improved outcome, though chemo-naive patients may be cured with chemotherapy alone [Papadimitris, 1997].

This ECC series of late relapses in testicular GCTs is limited to a small number of patients. The incidence rate of 0.85% is lower than in most other reported series [Baniel, 1995; Shahidi, 2002; Carver, 2005]. The ECC receives few extra-regional referrals, this therefore represents a true population-based incidence and is similar to the rate of 1.3% recently reported in a Norwegian cohort [Oldenburg, 2006]. As described in other published series, NSGCT was found to have a higher incidence of LR than seminoma. Four patients relapsed whilst on surveillance and 2 after a previous earlier relapse. It is of interest that no patients who presented with metastatic disease relapsed late. Unlike many other series in which tumour markers were raised in up to three-quarters of patients, αFP and β-hCG levels were normal at LR in all of these cases.

One patient had a resection specimen containing elements of adenocarcinoma. This is in line with a series published by Lutke Holzik et al. where 14% of recurrences contained non-germ cell malignancy [Lutke Holzik, 2003]. Sarcomas and adenocarcinomas were the most common pathologies encountered and complete surgical resection was recommended.

Following treatment for LR, all patients in this series remain free from recurrence after a median follow up of 52 months. Chemo-naive patients appear to have been successfully salvaged with chemotherapy alone and patients previously exposed to cisplatin-based chemotherapy have been salvaged with complete surgical excision. This is contrary to the experience of most other series where outcome following LR is poor. This may be because many of these patients had not previously been exposed to platinum-based chemotherapy, complete excisions were achieved where surgery was used, TD, a previously described good prognostic indicator, was a frequent pathological finding, most patients presented with single site late relapse and lastly follow-up time for some of the patients is limited.

## Conclusions

It is not clear what duration of follow-up is optimal for germ cell tumours of the testis. Some groups advise lifelong follow-up of all patients with malignant germ cell tumours of the testis though this is resource-intensive and the majority of patients do not relapse. The Royal Marsden advocates lifelong follow-up only in patients who initially presented with metastatic disease since this subgroup had a higher incidence of LR in their series [Shahidi, 2002]. Since very few patients with NSGCT relapse between 5 and 10 years there is some support for limiting follow-up to 5 years. In this cohort of 703 patients, one patient presented symptomatically greater than 10 years from primary treatment, two patients symptomatically within 5 years and two patients by protocolled CT surveillance within 5 years. Thus, only one patient (in whom CXR identified a mediastinal mass) was picked up by additional clinic follow-up between 5 and 10 years. Patient numbers in this series of Scottish patients are small and therefore definitive conclusions cannot be reached but in view of the valuable resources required for this additional follow-up (clinic time, x-ray performance and reporting time), unnecessary x-ray exposure and patient anxiety, on the basis of this small series the benefit of follow-up after five years is questionable. Risk factors for relapse including an earlier relapse and TD at resection should be taken into account when planning the duration of follow up. Whenever discharged there will always be a small risk of relapse and patients should be advised to present promptly should new symptoms develop.

## Figures and Tables

**Table 1:** Primary presentation and management.

Initial presentation						
Patient	1	2	3	4	5	6
Royal Marsden hospital stage	I	I	I	I	I	I
Initial pathology	MTI	Mixed	Seminoma	Seminoma	MTI	MTI
Markers elevated	αFP	βHCG	No	No	αFP	No
Treatment after orchidectomy	Surveillance	Surveillance	Surveillance	Para-aortic XRT	Surveillance	Surveillance

**Abbreviation:** MTI: Malignant teratoma intermediate.

**Table 2:** Late relapse presenting features initial management and management at relapse.

**Late relapse**						
Time to LR from 1° Treatment (months)	24	27	135	28	33	135
Time to LR from ER (months)	n.a.	n.a.	n.a.	n.a.	21	105
Mode of diagnosis	CT	CT	Clinical	Clinical	Clinical	CXR
Markers at LR	NEG	NEG	NEG	NEG	NEG	NEG
Site of LR	Para-aortic	Para-aortic	Para-aortic + Neck	Neck	Neck	Mediastinum
LR pathology	None	None	Seminoma	Seminoma	TD	TD with ADENOCA
LR treatment	Chemo	Chemo	Chemo	Surgery + Chemo	Surgery	Surgery
Response to treatment	CR	CR	CR	CR	CR	CR

**Outcome**						
Follow up (months)	54	136	50	90	20	30
Outcome	NED	NED	NED	NED	NED	NED

**Abbreviations:** n.a.: not applicable; NEG: Negative; TD: Teratoma Differentiated; MTI: malignant teratoma intermediate; CR: Complete Chemoresponse; NED: No evidence of disease.

**Table 3:** Literature summary.

	Dieckmann et al. 2005 [12]	George et al. 2003 [13]	Baniel et al. 1995 [8]	Shahidi et al. 2002 [11]	Ronnen et al. 2005 [14]	Gerl et al. 1997 [10]	Geldart et al. 2006 [8]	Detti et al. 2007
No of patients with LR	122	83	81	53	29	25	20	6
Incidence	n.a.	n.a.	2.9%	4.2%	n.a.	4.3%	n.a	0.85%
Median age	29	36	n.a.	n.a.	n.a.	n.a.	23	39
Median time to LR	4.1 years	7.1 years	6.2 years	n.a.	10.5 years	5.4 years	9 years	2.5 years
Marker positivity	αFP:52% βHCG:29%	αFP: 52% βHCG: 10% αFP + βHCG: 5%	αFP: 43% βHCG: 28% αFP + βHCG: 14%	n.a.	αFP:69% βHCG:21%	αFP:67% βHCG:8%	95% pos.	None pos.
Treatment at LR	Chemo alone: 45/122 (37%) Surgery alone: 13/122 (11%)	Chemo: 32/83 (39%) Surgery: 49/83 (59%)	Chemo alone: 65/81 (80%) Surgery alone: 16/81 (20%)	n.a.	Chemo: 29/29 (100%) Surgery: 12/29 (41%)	Chemo: 20/25 (80%) Surgery alone: 3/25 (12%)	75% surgery 25% chemo	50% chemo 30% surgery 20% combined
Chemotherapy complete response rate	n.a.	CR: 6/32 (19%)	CR:17/65 (26%)	n.a.	CR: 7/29 (24%)	CR: 12/20 (60%)	None	100%
Disease-free rates	77/120 (64%)	38/83 (46%)	21/81 (26%)	69%	8/29 (28%)	9/25 (36%)	80%	100%
Median follow up	n.a.	2.1 years	4.8 years	10.2 years	4.2 years	3.2 years	42 months	52 months
